# Contrasting roles of PSGL-1 and PD-1 in regulating T-cell exhaustion and function during chronic viral infection

**DOI:** 10.1128/jvi.02242-24

**Published:** 2025-02-06

**Authors:** Karla M. Viramontes, Melissa N. Thone, Jamie-Jean De La Torre, Emily N. Neubert, Julia M. DeRogatis, Chris Garcia, Monique L. Henriquez, Roberto Tinoco

**Affiliations:** 1 Department of Molecular Biology and Biochemistry, Charlie Dunlop School of Biological Sciences, University of California Irvine 209685 , Irvine, California, USA; 2 Center for Virus Research, University of California Irvine 453142 , Irvine, California, USA; 3 Institute for Immunology, University of California Irvine 8788 , Irvine, California, USA; 4 Chao Family Comprehensive Cancer Center, University of California Irvine 8788 , Irvine, California, USA; St. Jude Children's Research Hospital, Memphis, Tennessee, USA

**Keywords:** T cells, immune dysfunction, CD8 T cell, viral immunity, lymphocytic choriomeningitis virus

## Abstract

Immune checkpoints are critical regulators of T-cell exhaustion, impairing their ability to eliminate antigens present during chronic viral infections. Current immune checkpoint inhibitors (ICIs) used in the clinic aim to reinvigorate exhausted T cells; yet, most patients fail to respond or develop resistance to these therapies, underscoring the need to better understand these immunosuppressive pathways. PSGL-1 (*Selplg*), a recently discovered immune checkpoint, negatively regulates T-cell function. We investigated the cell-intrinsic effects of PSGL-1, PD-1, and combined deletion on CD8^+^ T cells during chronic viral infection. We found that combined PSGL-1 and PD-1 (*Selplg^-/-^Pdcd1^-/-^*) deficiency in CD8^+^ T cells increased their frequencies and numbers throughout chronic infection compared to the wild type. This phenotype was primarily driven by PD-1 deficiency. Furthermore, while PD-1 deletion increased virus-specific T-cell frequencies, it was detrimental to their function. Conversely, PSGL-1 deletion improved T-cell function but resulted in lower frequencies and numbers. The primary mechanism behind these differences in cell maintenance was driven by proliferation rather than survival. Combined PSGL-1 and PD-1 deletion resulted in defective T-cell differentiation, driving cells from a progenitor self-renewal state to a more terminal dysfunctional state. These findings suggest that PD-1 and PSGL-1 have distinct, yet complementary, roles in regulating T-cell exhaustion and differentiation during chronic viral infection. Overall, this study provides novel insights into the individual and combined roles of PSGL-1 and PD-1 in CD8^+^ T-cell exhaustion. It underscores the potential of targeting these checkpoints in a more dynamic and sequential manner to optimize virus-specific T-cell responses, offering critical perspectives for improving therapeutic strategies aimed at reinvigorating exhausted CD8^+^ T cells.

**IMPORTANCE:**

Our findings provide a comprehensive analysis of how the dual deletion of PD-1 and PSGL-1 impacts the response and function of virus-specific CD8^+^ T cells, revealing novel insights into their roles in chronic infection. Notably, our findings show that while PD-1 deletion enhances T-cell frequencies, it paradoxically reduces T-cell functionality. Conversely, PSGL-1 deletion improves T-cell function but reduces their survival. Whereas the combined deletion of PSGL-1 and PD-1 in CD8^+^ T cells improved their survival but decreased their function and progenitor-exhausted phenotypes during infection. We believe our study advances the understanding of immune checkpoint regulation in chronic infections and has significant implications for developing more effective immune checkpoint inhibitor (ICI) therapies.

## INTRODUCTION

CD8^+^ T cells are critical lymphocytes responsible for the effective elimination of pathogens. In acute viral infections, anti-viral CD8^+^ T cells rapidly develop effector functions and eradicate the pathogen. However, in chronic infections such as Hepatitis B and C, and human immunodeficiency virus (HIV), the virus persists at high levels (1). In these chronic settings where antigen persists and there is continuous immunosuppression, anti-viral T cells become progressively more exhausted. In this dysfunctional state, T cells undergo changes in transcriptional and epigenetic programs resulting in various states of exhaustion (2). T-cell exhaustion is characterized by the progressive loss of cytokine production, cytotoxic ability, decreased survival and proliferative potential, and ultimately in apoptosis (1, 3, 4). Furthermore, as T cells differentiate into more dysfunctional states, inhibitory receptors such as PD-1 and CTLA-4, among many others, are upregulated (5, 6). These increased levels of inhibitory receptors are sustained and promote further T-cell dysfunction by dampening TCR signaling (7). Exhausted T cells are heterogeneous and can be separated into ever-increasing distinct subsets including progenitor (TEXprog), progenitor-like (TEXproglike), effector (TEXeff), and terminal exhausted T cells (TEXterm) (8, 9). TEXprog can self-renew, have high proliferative potential, and differentiate along the spectrum of exhaustion into TEXterm. TEXterm are the most exhausted but retain sufficient function to keep viral replication under control (8). However, TEXterm are ultimately lost by apoptosis. Provided the important and different roles of exhausted T-cell subsets, there is a critical need to better understand their biology. Collectively, the ability to reinvigorate exhausted T cells is of great therapeutic interest.

Immune checkpoint inhibitors (ICIs) have revolutionized immunotherapy through their ability to reinvigorate exhausted T cells, demonstrating efficacy in the clinic in patients with chronic viral infections and cancer (10–13). This is primarily attributed to the enhanced expansion of TEXprog cells that give rise to T cells with enhanced effector functions (8). This re-expansion facilitates viral clearance. Current FDA-approved ICIs include monoclonal antibodies targeting PD-1, PD-L1, and CTLA-4, which restore exhausted T cells into a more effector state (14). Moreover, combination treatment with multiple ICIs further enhances the immune response in these diseases (12, 13). While ICIs are now considered the standard of care for multiple cancers, very few patients respond to either single or combination ICI treatment (15). In addition, initial responders commonly develop resistance (16). Some studies have suggested that a lack of response to ICI treatment is partially due to the significantly changed epigenetic profile of exhausted T cells, making it unfeasible to revert epigenetically to an effector state, a term described as “epigenetic scarring” (17–19). ICIs are primarily thought to work by improving T-cell responses and understanding how they impact T cells intrinsically in terms of their differentiation and function is clinically relevant. In addition, because resistance to treatment is common, there is great interest in identifying and targeting novel immune checkpoints alone or in combination with FDA-approved ICIs.

P-selectin glycoprotein ligand-1 (PSGL-1, *Selplg*) is a transmembrane protein expressed on all hemopoietic cell subsets, including myeloid and lymphoid cells. Although originally discovered in neutrophils for their role in adhesion and migration through selectin engagement, studies have also identified PSGL-1 as an immune checkpoint on T cells (5, 20). PSGL-1 was first observed as a negative regulator of T-cell function in PSGL-1 knockout (*Selplg^-/-^*) mice infected with either acute lymphocytic choriomeningitis virus strain Armstrong (LCMV ARM) or chronic lymphocytic choriomeningitis virus strain Clone 13 (LCMV Cl13). Deficiency in PSGL-1 resulted in increased frequencies of virus-specific effector and memory T cells in an acute infection setting and increased virus-specific T-cell frequencies, improved T-cell function, reduced expression of inhibitory receptors, and reduced viremia in a chronic infection setting (5, 21). More recently, our group investigated the cell-intrinsic role of PSGL-1 in exhausted T-cell function and differentiation using a genetic deletion model. We observed that deletion of PSGL-1 in anti-viral CD8^+^ T cells reduced their frequencies at late stages of chronic viral infection but greatly increased their effector functions (22). We further observed that PSGL-1 deletion in combination with PD-L1 blockade increased T-cell frequencies and enhanced effector functions, suggesting potential synergism between these two immune checkpoints (22). While these studies began to reveal the intricacies between PSGL-1 and PD-1, the mechanisms by which these two immune checkpoints impact exhausted T-cell differentiation, maintenance, and function intrinsically remained to be elucidated.

In this study, we investigated the cell-intrinsic roles of dual PSGL-1 and PD-1 genetic deletion, in the response of exhausted anti-viral P14 CD8^+^ T cells. We found that relieving PSGL-1 and PD-1 immune checkpoints increased and sustained frequencies of *Selplg^-/-^Pdcd1^-/-^* P14 CD8^+^ T cells. The maintenance of these cells was due to increased proliferation and survival relative to WT P14 CD8^+^ T cells. Unexpectedly, we observed decreased cytokine production and degranulation ability in *Selplg^-/-^Pdcd1^-/-^* P14 CD8^+^ T cells at both early and late stages of chronic viral infection, suggesting an important role for these immune checkpoints in preserving T-cell functionality. Furthermore, *Selplg^-/-^Pdcd1^-/-^* P14 CD8^+^ T cells exhibited decreased TCF-1 and KLRG-1 expression and increased expression of TOX, TIGIT, LAG-3, and CD101. Finally, and consistent with these results, *Selplg^-/-^Pdcd1^-/-^* P14 CD8^+^ T cells had reduced frequencies of SLAMF6^+^CX3CR1^-^ TEXprog cells and increased frequencies of SLAMF6^-^CX3CR1^-^ TEXterm cells both at early and late stages of infection, suggesting that PSGL-1 and PD-1 deletion favor a more terminally exhausted T-cell fate. Our findings highlight the important and different functions of PSGL-1 and PD-1 in anti-viral CD8^+^ T cells and how they regulate the expansion, maintenance, and function of exhausted T cells. Collectively, our findings provide a better understanding of the roles that these immune checkpoints play and provide potential future directions to best harness the unique benefits of these ICIs.

## RESULTS

### Virus-specific *Selplg^-/-^Pdcd1^-/-^* CD8^+^ T cells expand and are maintained at higher frequencies during chronic infection

**Fig 1:**
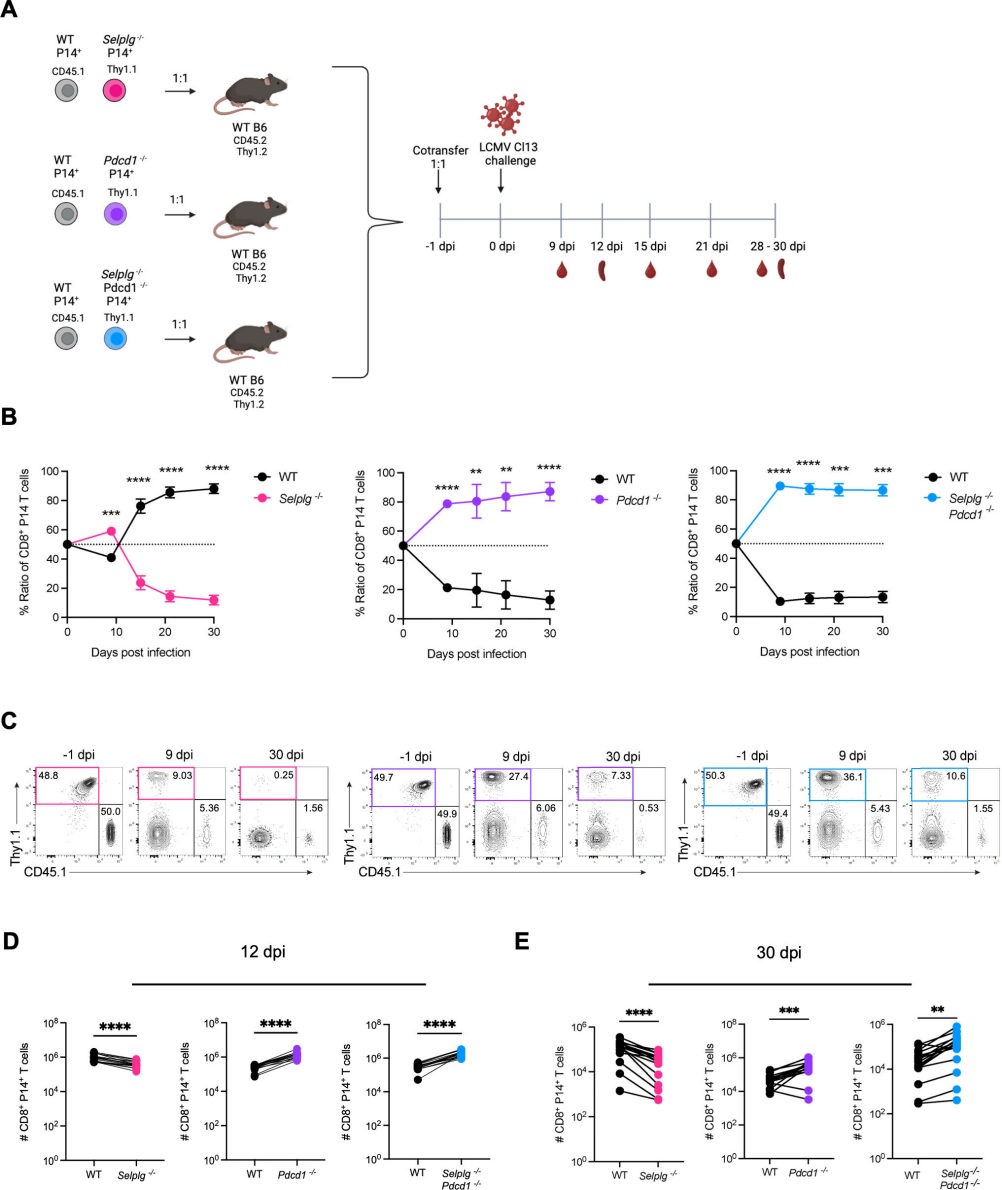
*Selplg^-/-^Pdcd1^-/-^* P14 CD8^+^ T cells expand and are sustained over the course of chronic viral infection. (**A**) WT and either *Selplg^-/-^*, *Pdcd1^-/-^*, or *Selplg^-/-^Pdcd1^-/-^* P14 CD8^+^ T cells were adoptively co-transferred at a 1:1 ratio into WT recipients and infected with Cl13 one day after. (**B**) Percent ratios of WT and either *Selplg^-/-^*, *Pdcd1^-/-^*, or *Selplg^-/-^Pdcd1^-/-^* P14 CD8^+^ T cells in the blood at the indicated timepoints. (**C**) Representative flow cytometry plots of WT and *Selplg^-/-^*, *Pdcd1^-/-^*, or *Selplg^-/-^Pdcd1^-/-^* P14 CD8^+^ T cells at −1 dpi, 9 dpi, and 30 dpi in the blood. (**D**) Numbers of WT and either *Selplg^-/-^*, *Pdcd1^-/-^*, or *Selplg^-/-^Pdcd1^-/-^* P14 CD8^+^ T cells at 12 dpi and (**E**) 30 dpi in the spleen. *P < 0.05, **P < 0.01, ***P < 0.001 (paired t-test). Data are representative of five independent experiments all with five or more mice per group (error bars, s.e.m.).

Our previous study revealed that virus-specific *Selplg^-/-^* CD8^+^ T cells expand to greater frequencies than WT T cells with PD-L1 blockade at late stages of chronic LCMV Cl13 infection (22). Therefore, we next wanted to investigate the cell-intrinsic role of PD-1 and PSGL-1 in CD8^+^ T cells over the course of chronic viral infection. We adoptively co-transferred small numbers (1 × 10^3^) of TCR transgenic (Tg) WT P14 (CD45.1^+^) and *Selplg^-/-^Pdcd1^-/-^* double knockout (Thy1.1^+^) P14 CD8^+^ naïve T cells into WT (CD45.2^+^Thy1.2^+^) hosts at a 1:1 ratio. Naïve WT P14 and *Selplg^-/-^* (Thy1.1^+^) or *Pdcd1^-/-^* (Thy1.1^+^) single knockout P14 CD8^+^ T cells were also adoptively co-transferred into WT hosts at a 1:1 ratio as controls (Fig. 1A). One day later, mice were infected with LCMV Cl13 and the T-cell response was analyzed at 9, 15, 21, and 30 days post-infection (dpi) in the blood. Like our previous study, we observed increased ratios and frequencies of *Selplg^-/-^* P14 CD8^+^ T cells compared to WT at 9dpi, at a 60:40 ratio, respectively. This initial expansion was followed by a decrease in *Selplg^-/-^* P14 CD8^+^ T-cell frequencies and ratios up to 30 dpi compared to WT (Fig. 1B and C). Like *Selplg^-/-^* P14 CD8^+^ T cells, *Pdcd1^-/-^* P14 CD8^+^ T cells initially expanded to higher ratios and frequencies at 9 dpi, albeit more dramatically at an 80:20 ratio, respectively. Conversely, *Pdcd1^-/-^* P14 CD8^+^ T cells were maintained at higher frequencies and ratios than WT up to 30 dpi (Fig. 1B and C). Interestingly, we also observed higher ratios and frequencies of *Selplg^-/-^Pdcd1^-/-^* P14 T cells compared to WT at 9 dpi at a 90:10 ratio, respectively. This increase was maintained throughout infection up to 30 dpi (Fig. 1B and C). Similar to the trends observed in the blood, we observed decreased numbers of *Selplg*^-/-^ and increased numbers of *Pdcd1^-/-^* and *Selplg^-/-^Pdcd1^-/-^* P14 CD8^+^ T cells in the spleen relative to WT at both 12 and 30 dpi (Fig. 1D and E). These findings highlight immune checkpoint differences and show that while PSGL-1 is necessary to maintain T cells, PD-1 deficiency, and PSGL-1 with PD-1 inhibition improve T-cell maintenance during chronic viral infection.

### Effector function of virus-specific *Selplg^-/-^Pdcd1^-/-^* CD8^+^ T cells is decreased early during viral infection

**Fig 2:**
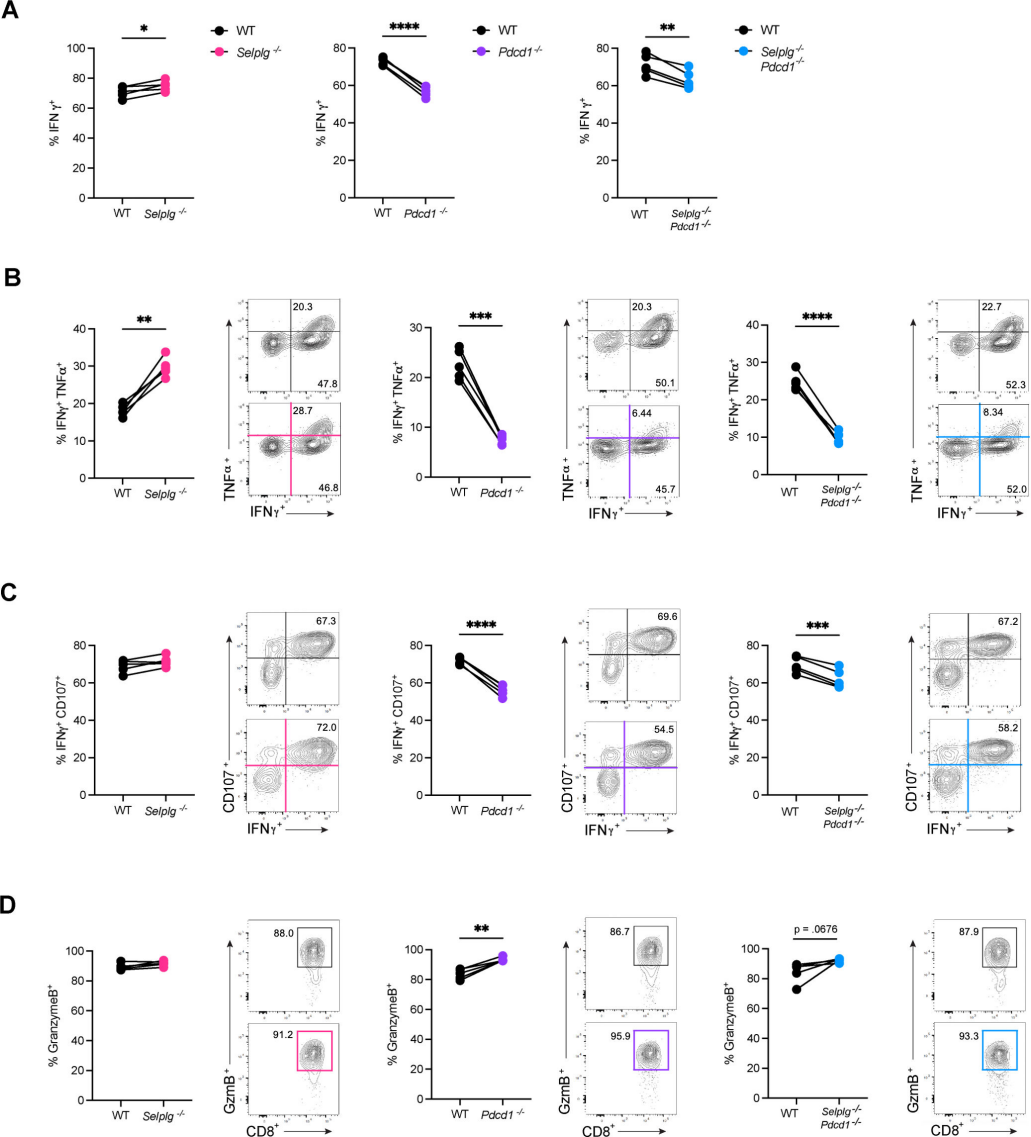
*Selplg^-/-^Pdcd1^-/-^* P14 CD8^+^ T cells exhibit decreased effector function and cytotoxicity during the early stages of chronic viral infection. Blood was isolated from Cl13-infected mice at 9dpi and stimulated with GP_33-41_ cognate peptide ex vivo. Frequencies and representative flow cytometry plots of (**A**) IFNγ^+^, (**B**) IFNγ^+^ TNF-α^+^, (**C**) IFNγ^+^CD107a^+^, and (**D**) Granzyme B^+^ in WT and *Selplg^-/-^*, *Pdcd1^-/-^*, or *Selplg^-/-^Pdcd1^-/-^* P14 CD8^+^ T cells. *P < 0.05, **P < 0.01, ***P < 0.001 (paired t-test). Data are representative of five independent experiments all with five or more mice per group (error bars, s.e.m.).

We next characterized the functionality and cytotoxicity of the adoptively transferred Tg T cells during the early stages of chronic viral infection at 9dpi in the blood. As in our previous study, we observed an increase in the frequencies of *Selplg^-/-^* P14 CD8^+^ T cells producing interferon-gamma (IFNγ) compared to WT (Fig. 2A). By contrast, and as previously documented, significantly lower frequencies of *Pdcd1^-/-^* P14 CD8^+^ T cells produced IFNγ compared to WT (Fig. 2A). In *Selplg^-/-^Pdcd1^-/-^* P14 CD8^+^ T cells, we also observed significantly decreased frequencies in IFNγ production compared to WT (Fig. 2A). We next looked at the polyfunctionality of these exhausted T cells. Compared to their WT counterparts, significantly higher frequencies of *Selplg^-/-^* P14 CD8^+^ T cells produced both IFNγ and tumor necrosis factor-alpha (TNF-α), while significantly lower frequencies of *Pdcd1^-/-^* P14 CD8^+^ T cells produced both inflammatory cytokines (Fig. 2B). Importantly, IFNγ and TNF-α production were also significantly reduced in *Selplg^-/-^Pdcd1^-/-^* P14 CD8^+^ T cells compared to WT (Fig. 2B). Another characteristic of T-cell exhaustion is their decreased cytotoxic ability. Therefore, we assessed the generation of IFNγ in combination with the expression of CD107a, a surface marker for degranulation. As previously noted, we observed slightly increased frequencies of IFNγ^+^CD107a^+^ *Selplg^-/-^* P14 CD8^+^ T cells compared to WT at 9 dpi (Fig. 2C). By contrast, we observed significantly lower IFNγ^+^CD107a^+^*Pdcd1^-/-^* P14 CD8^+^ T-cell frequencies compared to their WT counterparts (Fig. 2C). *Selplg^-/-^Pdcd1^-/-^* P14 CD8^+^ T cells also exhibited significantly reduced IFNγ^+^CD107a^+^ frequencies compared to WT (Fig. 2C). Lastly, we investigated the ability of these exhausted T cells to produce granzyme B. We saw no differences in granzyme B production between WT and *Selplg^-/-^* P14 CD8^+^ T cells and observed increased frequencies of *Pdcd1^-/-^* P14 CD8^+^ T cells producing granzyme B compared to their WT counterparts (Fig. 2D). Interestingly, we observed only a subtle increase in granzyme B^+^ frequencies in *Selplg^-/-^Pdcd1^-/-^* P14 CD8^+^ T compared to WT (Fig. 2D). These findings show that while PSGL-1 hinders T-cell function, PD-1 alone and PD-1 with PSGL-1 play an important and protective role in CD8^+^ T-cell function.

### Virus-specific *Selplg^-/-^Pdcd1^-/-^* CD8^+^ T cells develop a terminal fate at early stages of chronic infection

**Fig 3:**
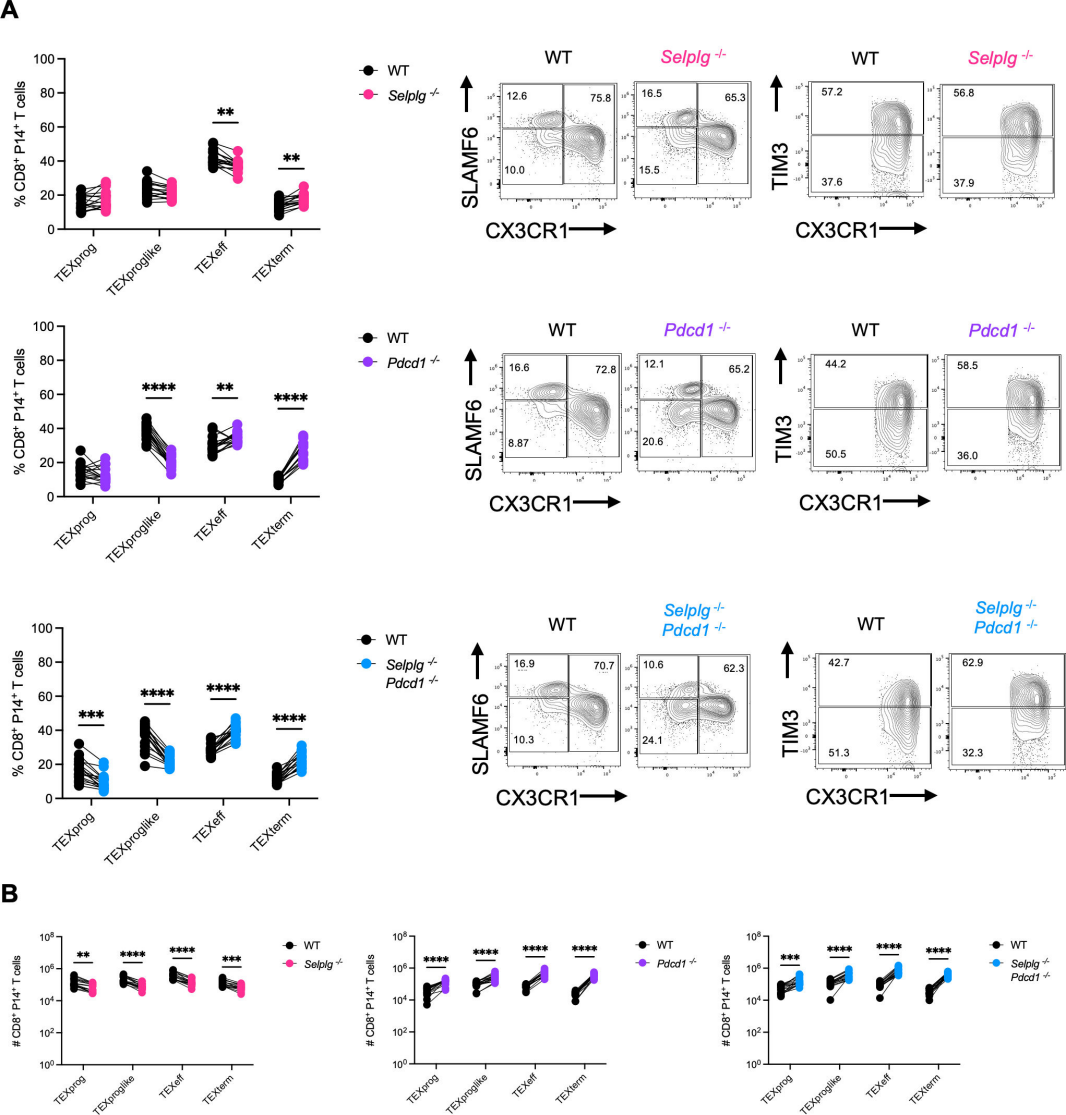
*Selplg^-/-^Pdcd1^-/-^* P14 CD8^+^ T cells exhibit a more terminal phenotype during chronic viral infection. Spleens were isolated from Cl13-infected mice at 12 dpi. (**A**) Frequencies and representative flow cytometry plots of SLAMF6^+^CX3CR1^-^ progenitor exhausted (TEXprog), CX3CR1^+^TIM3^-^ progenitor exhausted like (TEXproglike), CX3CR1^+^TIM3^+^ effector exhausted (TEXeff), and SLAMF6^-^CX3CR1^-^ terminal exhausted (TEXtem) between WT and *Selplg^-/-^*, *Pdcd1^-/-^*, or *Selplg^-/-^Pdcd1^-/-^* P14 CD8^+^ T cells. (**B**) Numbers of TEXprog, TEXproglike, TEXeff, and TEXtem between WT and either *Selplg^-/-^*, *Pdcd1^-/-^*, or *Selplg^-/-^Pdcd1^-/-^* P14 CD8^+^ T cells. *P < 0.05, **P < 0.01, ***P < 0.001 (paired t-test). Data are representative of two combined independent experiments all with five or more mice per group (error bars, s.e.m.).

We further characterized the exhaustion subsets of co-transferred cells at 12 dpi. P14 CD8^+^ T cells were stratified into progenitor exhausted (TEXprog; SLAMF6^+^CX3CR1^−^), progenitor exhausted-like (TEXproglike; CX3CR1^+^TIM3^−^), effector exhausted (TEXeff; CX3CR1^+^TIM3^+^), or terminally exhausted (TEXterm; SLAMF6^−^CX3CR1^−^) subsets. *Selplg^-/-^* P14 CD8^+^ T cells had similar frequencies of TEXprog and TEXproglike cells, slightly decreased frequencies of TEXeff cells, and slightly increased frequencies of TEXterm cells relative to WT (Fig. 3A). *Pdcd1^-/-^* P14 CD8^+^ T cells had similar frequencies of TEXprog with significantly decreased TEXproglike cells accompanied by significantly increased frequencies of TEXeff and TEXterm cells relative to WT (Fig. 3A). *Selplg^-/-^Pdcd1^-/-^* P14 CD8^+^ T cells had significantly decreased frequencies of TEXprog and TEXproglike cells accompanied by significantly increased frequencies of TEXeff cells and TEXterm cells relative to WT (Fig. 3A). Furthermore, numerically, *Selplg^-/-^* P14 CD8^+^ T-cell subsets were all lower compared to WT (Fig. 3B), whereas all subsets of *Pdcd1^-/-^* and *Selplg^-/-^Pdcd1^-/-^* P14 CD8^+^ T cells were higher relative to WT (Fig. 3B). These data showed that combined PSGL-1 and PD-1 deletion increased virus-specific T-cell numbers with a terminal phenotype.

### Virus-specific *Selplg^-/-^Pdcd1^-/-^* CD8^+^ T cells expand and persist by increased proliferation and enhanced survival

**Fig 4:**
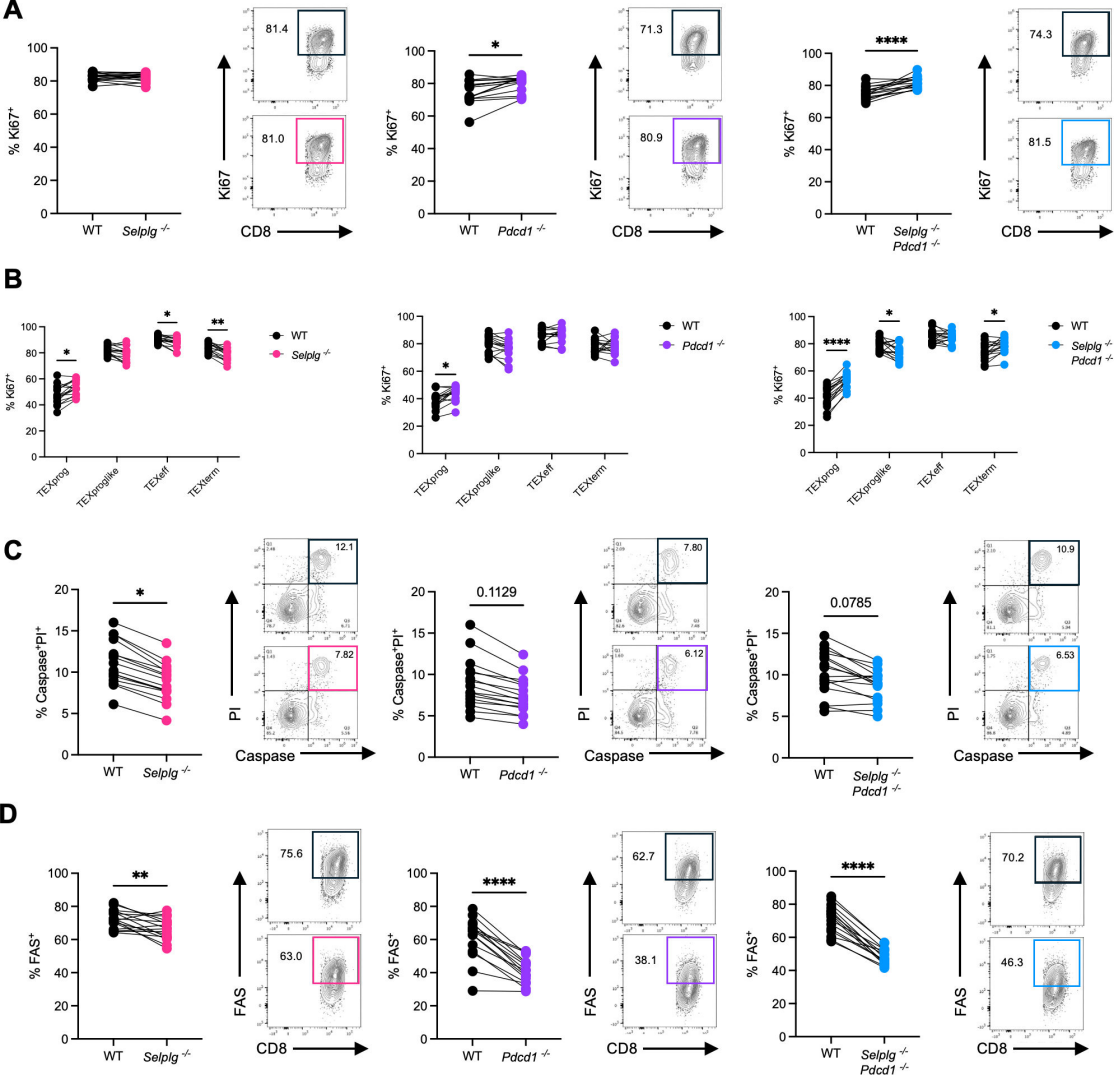
*Selplg^-/-^Pdcd1^-/-^* P14 CD8^+^ T-cell expansion is due to increased proliferation and improved survival. Spleens were isolated from Cl13-infected mice at 12 dpi. (**A**) Frequencies and representative flow cytometry plots of Ki67^+^ cells between WT and either *Selplg^-/-^*, *Pdcd1^-/-^*, or *Selplg^-/-^Pdcd1^-/-^* P14 CD8^+^ T cells. (**B**) Frequencies of Ki67^+^ cells within SLAMF6^+^CX3CR1^-^ progenitor exhausted (TEXprog), CX3CR1^+^TIM3^-^ progenitor exhausted like (TEXproglike), CX3CR1^+^TIM3^+^ effector exhausted (TEXeff), and SLAMF6^-^CX3CR1^-^ terminal exhausted (TEXtem) between WT and *Selplg^-/-^*, *Pdcd1^-/-^*, or *Selplg^-/-^Pdcd1^-/-^* P14 CD8^+^ T cells. (**C**) Frequencies and representative flow cytometry plots of late apoptotic Caspase^+^ PI^+^ cells between WT and *Selplg^-/-^*, *Pdcd1^-/-^*, or *Selplg^-/-^Pdcd1^-/-^* P14 CD8^+^ T cells. (**D**) Frequencies and representative flow cytometry plots of FAS^+^ expressing cells between WT and *Selplg^-/-^*, *Pdcd1^-/-^*, or *Selplg^-/-^Pdcd1^-/-^* P14 CD8^+^ T cells. *P < 0.05, **P < 0.01, ***P < 0.001 (paired t-test). Data are representative of two combined independent experiments all with five or more mice per group (error bars, s.e.m.).

We next sought to understand the mechanism behind the loss of *Selplg^-/-^* but the persistence of *Pdcd1^-/-^*, and *Selplg^-/-^Pdcd1^-/-^* P14 CD8^+^ T cells by evaluating their changes in proliferation and survival. *Selplg ^-/-^* P14 CD8^+^ T cells had similar rates of proliferation while *Pdcd1^-/-^* and *Selplg^-/-^Pdcd1^-/-^* P14 CD8^+^ T cells showed increased proliferation relative to WT at 12 dpi (Fig. 4A). Within subsets, TEXprog showed increased proliferation in *Selplg^-/-^*, *Pdcd1^-/-^*, and *Selplg^-/-^Pdcd1^-/-^* P14 CD8^+^ T cells relative to WT (Fig. 4B). This was expected as progenitor cells are known to respond by proliferating to ICI treatment (8). Interestingly, while *Selplg^-/-^* P14 CD8^+^ T cells with a terminal fate (TEXeff and TEXterm) had decreased proliferation, these cell subsets were proliferating at similar or higher rates in *Pdcd1^-/-^*, and *Selplg^-/-^Pdcd1^-/-^* P14 CD8^+^ T cells compared to WT (Fig. 4B). Provided that CD8^+^ T cells by 12 dpi are largely differentiated into effectors or terminal cell subsets, the higher proliferation in these subsets likely contributes to the higher frequencies and numbers observed in *Pdcd1^-/-^*, and *Selplg^-/-^Pdcd1^-/-^* P14 CD8^+^ T cells. These data demonstrated that part of the mechanism behind the increased CD8^+^ T cells lacking PD-1 or PSGL-1/PD-1 expression is due to sustained proliferation by their terminal exhausted cell subsets.

We next investigated whether changes in survival could account for the differences in the accumulation of responding T cells. Notably, *Selplg^-/-^* P14 CD8^+^ T cells had slightly lower Caspase^+^ PI^+^ cells compared to WT, while no differences were observed in *Pdcd1^-/-^*, and *Selplg^-/-^Pdcd1^-/-^* P14 CD8^+^ T cells relative to WT (Fig. 4C). Interestingly, Caspase^+^ PI^+^ frequencies in progenitor and terminal cells subsets were lower in all genotypes compared to WT (Fig. S1A). We further evaluated FAS (CD95) levels as expression by activated CD8^+^ T cells can induce their apoptosis through FASL engagement (23). Total co-transferred *Selplg^-/-^*, *Pdcd1^-/-^*, and *Selplg^-/-^Pdcd1^-/-^* P14 CD8^+^ T cells showed decreased frequencies of FAS^+^ cells relative to WT (Fig. 4D). However, increased frequencies of FAS^+^ cells were present in *Selplg^-/-^* P14 CD8^+^ T cells in comparison to *Pdcd1^-/-^* and *Selplg^-/-^Pdcd1^-/-^* P14 CD8^+^ T cells (Fig. 4D). Decreased frequencies of FAS^+^ cells were also observed within the majority of progenitor and terminal exhausted subsets (Fig. S1B). Decreased FAS^+^ cells in *Pdcd1^-/-^* and *Selplg^-/-^Pdcd1^-/-^* P14 CD8^+^ T cells when compared to *Selplg^-/-^* cells could contribute to the enhanced survival and maintenance of PD-1 and PSGL-1/PD-1-deficient CD8^+^ T cells (Fig. 4D). Importantly, this extended survival could also contribute to enhanced immunopathology commonly observed in LCMV Cl13 models in PD-1-deficient mice or mice treated with anti-PD-1/PD-L1 (24–26). Collectively, these data demonstrated that the loss of PSGL-1 deficient CD8^+^ T cells was driven by decreased proliferation of terminal cells, while the persistence of PD-1 and PSGL-1/PD-1 dual-deficient CD8^+^ T cells was driven by increased proliferation and survival of terminal-phenotype exhausted cells.

### Virus-specific *Selplg^-/-^Pdcd1^-/-^* CD8^+^ T cells have a terminal phenotype at late stages of chronic infectionte

**Fig 5:**
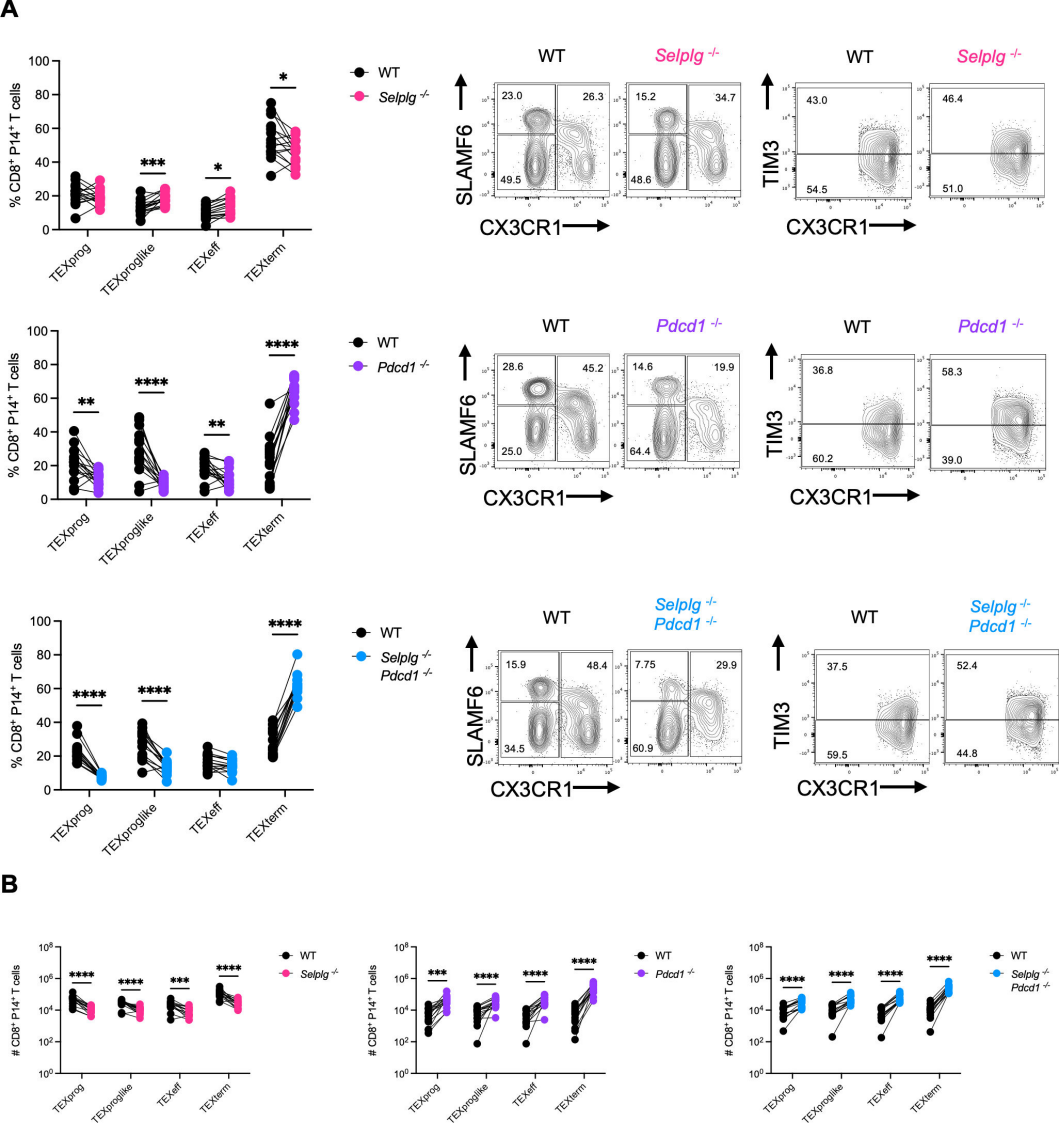
*Selplg^-/-^Pdcd1^-/-^* P14 CD8^+^ T cells exhibit a more terminal phenotype at late stages of chronic viral infection. Spleens were isolated from Cl13-infected mice at 28 dpi. (**A**) Frequencies and representative flow cytometry plots of SLAMF6^+^CX3CR1^−^ progenitor exhausted (TEXprog), CX3CR1^+^TIM3^−^ progenitor exhausted like (TEXproglike), CX3CR1^+^TIM3^+^ effector exhausted (TEXeff), and SLAMF6^−^CX3CR1^−^ terminal exhausted (TEXtem) between WT and either *Selplg^-/-^*, *Pdcd1^-/-^*, or *Selplg^-/-^Pdcd1^-/-^* P14 CD8^+^ T cells. (**B**) Numbers of TEXprog, TEXproglike, TEXeff, and TEXtem between WT and *Selplg^-/-^*, *Pdcd1^-/-^*, or *Selplg^-/-^Pdcd1^-/-^* P14 CD8^+^ T cells. *P < 0.05, **P < 0.01, ***P < 0.001 (paired t-test). Data are representative of two combined independent experiments all with five or more mice per group (error bars, s.e.m.).

We next characterized the exhaustion subsets of co-transferred cells in the spleen at 28 dpi. *Selplg^-/-^* P14 CD8^+^ T cells had similar frequencies of TEXprog, slightly increased frequencies of TEXproglike and TEXeff, and decreased frequencies of TEXterm relative to WT (Fig. 5A). *Pdcd1^-/-^* P14 CD8^+^ T cells had significantly decreased frequencies of TEXprog and TEXproglike cells, slightly decreased frequencies of TEXeff cells, and significantly increased frequencies of TEXterm cells relative to WT (Fig. 5A). Similarly, *Selplg^-/-^Pdcd1^-/-^* P14 CD8^+^ T cells had significantly decreased frequencies of TEXprog and TEXproglike cells, similar frequencies of TEXeff cells, but significantly increased frequencies of TEXterm cells relative to WT (Fig. 5A). These differences where progenitor exhausted cells were decreased while terminally exhausted cells increased in PSGL-1, PD-1, and PSGL-1/PD-1 knockout cells were also confirmed using TCF-1, SLAMF6, and TIM3 expression (Fig. S2A and B). By this late timepoint, we noted that numerically, all exhausted subsets were decreased in *Selplg^-/-^* T cells, whereas these numbers were significantly increased in *Pdcd1^-/-^* and *Selplg^-/-^Pdcd1^-/-^* P14 CD8^+^ T cells relative to WT (Fig. 5B). These findings indicated that in Cl13 infection, T cells deficient in PD-1 and PSGL-1/PD-1 had higher frequencies and numbers of terminally exhausted T cells. By contrast, T cells lacking only PSGL-1 expression did not maintain these exhausted subsets.

**Fig 6:**
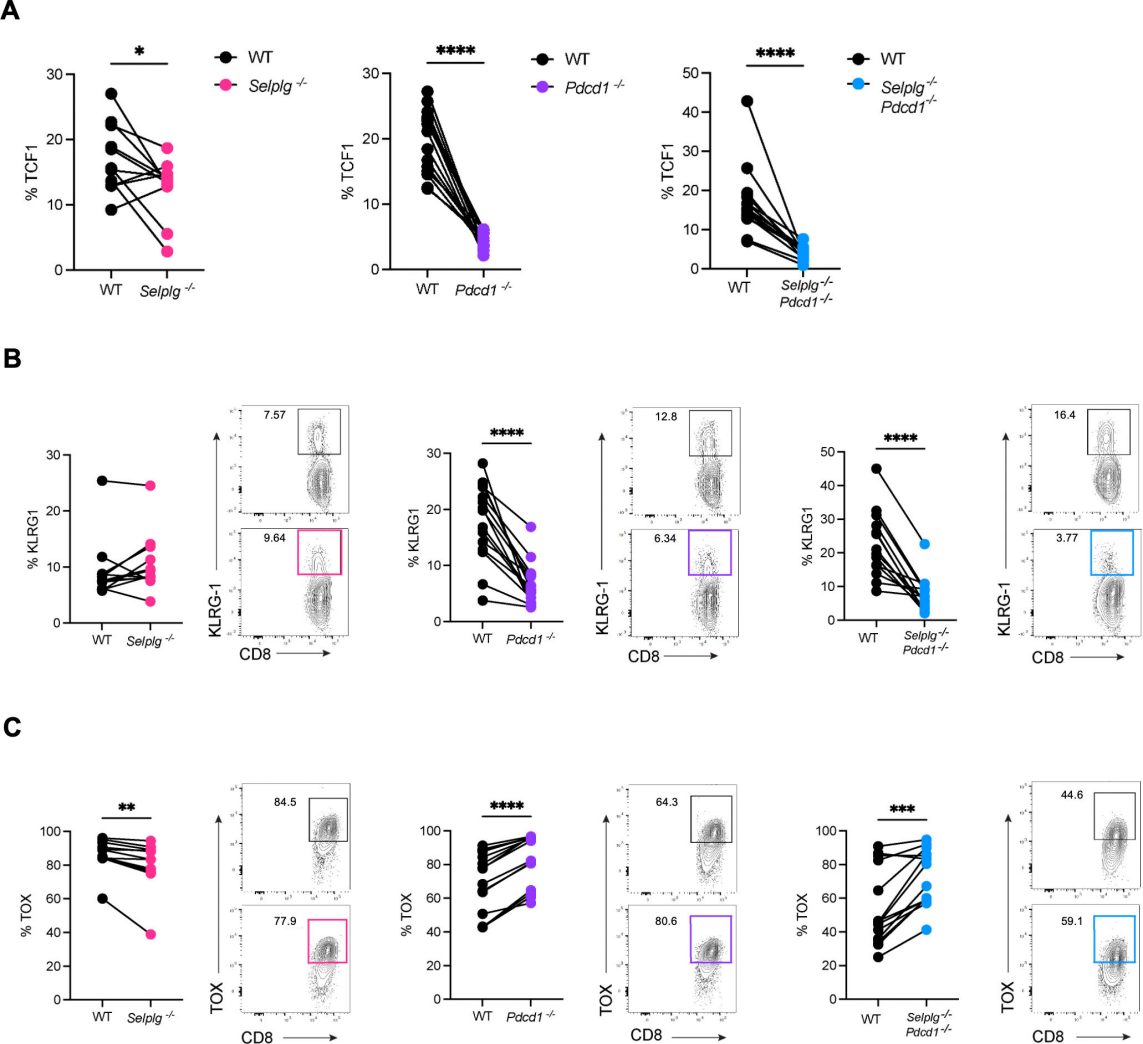
Expression of exhaustion markers in *Selplg^-/-^Pdcd1^-/-^* P14 CD8^+^ T cells. Spleens were isolated from Cl13-infected mice at 30 dpi. (**A**) Frequencies of TCF-1^+^cells between WT and *Selplg^-/-^*, *Pdcd1^-/-^*, or *Selplg^-/-^Pdcd1^-/-^* P14 CD8^+^ T cells. (**B**) Frequencies and representative flow cytometry plots of KLRG-1^+^ and (**C**) TOX^+^ between WT and *Selplg^-/-^*, *Pdcd1^-/-^*, or *Selplg^-/-^Pdcd1^-/-^* P14 CD8^+^ T cells. *P < 0.05, **P < 0.01, ***P < 0.001 (paired t-test). Data are representative of five independent experiments all with five or more mice per group (error bars, s.e.m.).

We evaluated TCF-1 and TOX transcription factor expression which are key in the differentiation of progenitor and terminal exhausted T cells (8, 9). While we observed decreased frequencies of TCF-1^+^ *Selplg^-/-^* P14 CD8^+^ T cells, there was a much more significant reduction in TCF-1^+^ cells in both *Pdcd1^-/-^* and *Selplg^-/-^Pdcd1^-/-^* P14 CD8^+^ T cells relative to WT (Fig. 6A). We next evaluated KLRG-1 expression since levels are decreased as T cells transition into terminally exhausted fates (27). There were no differences in KLRG-1 expression in *Selplg^-/-^* P14 CD8^+^ T cells compared to WT (Fig. 6B). However, we observed significantly lower frequencies of *Pdcd1^-/-^* and *Selplg^-/-^Pdcd1^-/-^* P14 CD8^+^ T cells expressing KLRG-1 compared to WT (Fig. 6B). We evaluated TOX expression and observed lower frequencies of TOX^+^ *Selplg^-/-^* P14 CD8^+^ T cells compared to WT (Fig. 6C). By contrast, we observed significantly higher frequencies of TOX^+^ *Pdcd1^-/-^* and *Selplg^-/-^Pdcd1^-/-^* P14 CD8^+^ T cells compared to WT (Fig. 6C). To further confirm the terminally exhausted phenotype, we investigated additional markers TIGIT, LAG-3, and CD101. We observed decreased frequencies and expression levels of TIGIT^+^ *Selplg^-/-^* P14 CD8^+^ T cells compared to WT (Fig. S3A). By contrast, we found significantly increased frequencies and expression levels of TIGIT^+^ *Pdcd1^-/-^* and *Selplg^-/-^Pdcd1^-/-^* P14 CD8^+^ T cells compared to WT (Fig. S3A). In *Selplg^-/-^*, *Pdcd1^-/-^*, and *Selplg^-/-^Pdcd1^-/-^* P14 CD8^+^ T cells, we observed decreased frequencies of LAG-3^+^ cells, but the LAG-3 expression levels were higher in LAG-3^+^ cells compared to WT (Fig. S3B). We noted decreased frequencies and expression levels of CD101^+^ *Selplg^-/-^* P14 CD8^+^ T cells (Fig. S3C). By contrast, we saw significantly increased frequencies and expression levels of CD101^+^ *Pdcd1^-/-^* and *Selplg^-/-^Pdcd1^-/-^* P14 CD8^+^ T cells compared to WT (Fig. S3C). Together, these findings further demonstrate that PSGL-1 deficiency maintains a more effector-like population while PD-1 deficiency drives cells to a more terminal fate.

### Virus-specific *Selplg^-/-^Pdcd1^-/-^* CD8^+^ T-cell function is decreased at late stages of chronic viral infection

**Fig 7:**
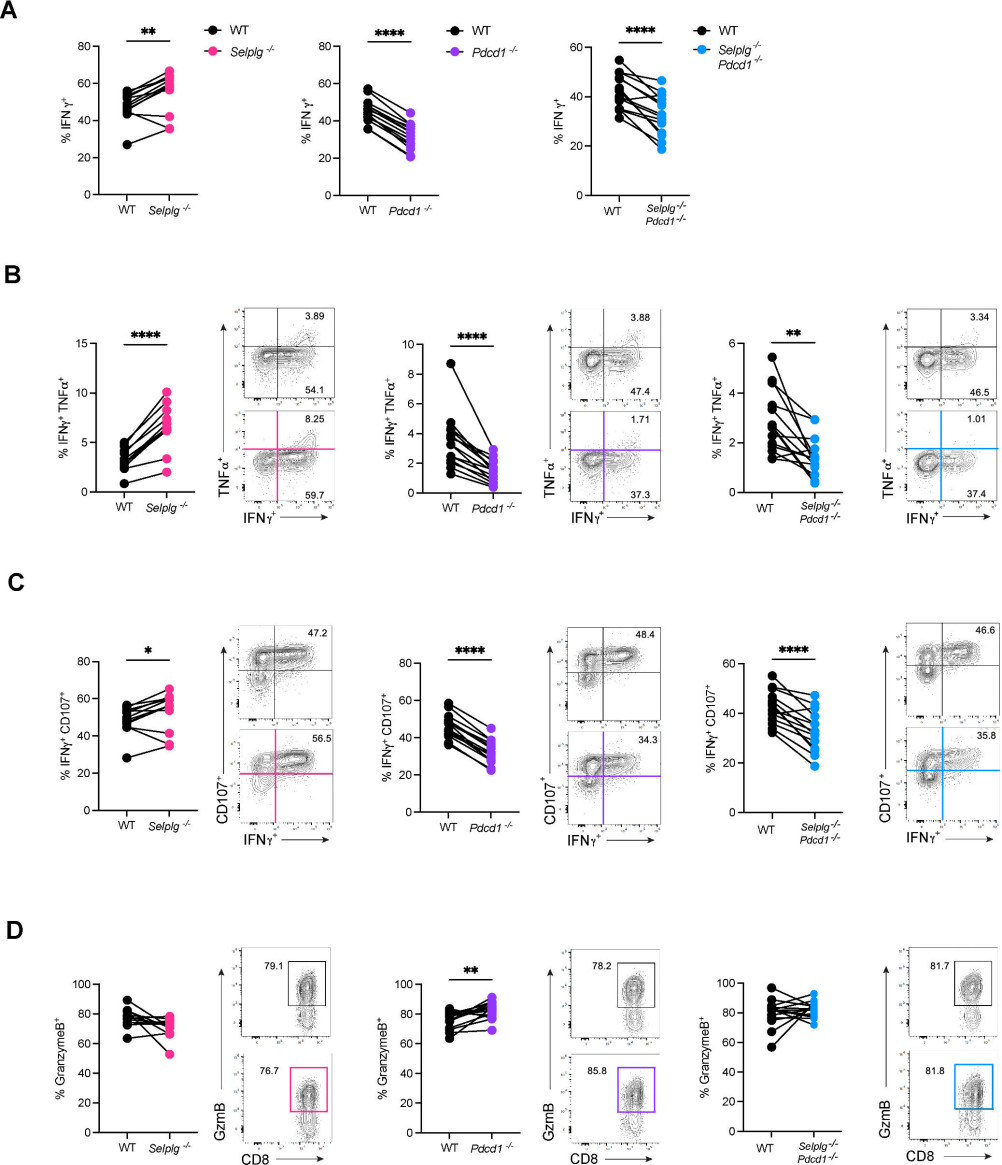
*Selplg^-/-^Pdcd1^-/-^* P14 CD8^+^ T cells exhibit decreased effector function and cytotoxicity at late stages of chronic viral infection. Spleens were isolated from Cl13-infected mice at 30 dpi and stimulated with GP_33-41_ cognate peptide *ex vivo*. Frequencies and representative flow cytometry plots of (**A**) IFNγ^+^, (**B**) IFNγ^+^ TNF-α^+^, (**C**) IFNγ^+^CD107a^+^, and (**D**) Granzyme B^+^ in WT and *Selplg^-/-^*, *Pdcd1^-/-^*, or *Selplg^-/-^Pdcd1^-/-^* P14 CD8^+^ T cells. *P < 0.05, **P < 0.01, ***P < 0.001 (paired t-test). Data are representative of five independent experiments all with five or more mice per group (error bars, s.e.m.)

During the early stages of infection, we observed increased function in *Selplg^-/-^* P14 CD8^+^ T cells and decreased function in *Pdcd1^-/-^* and *Selplg^-/-^Pdcd1^-/-^* P14 CD8^+^ T cells. To assess whether these differences were maintained over the course of infection, we tested the functional and cytotoxic capacity of co-transferred cells at 30 dpi in the spleen. As reported prior (22), and similar to trends observed at 9 dpi, we observed significant increases in the frequencies of IFNγ^+^ *Selplg^-/-^* and significantly decreased frequencies of IFNγ^+^ *Pdcd1 ^-/-^* P14 CD8^+^ T cells compared WT (Fig. 7A). Furthermore, we also observed significantly decreased frequencies of IFNγ^+^ *Selplg^-/-^Pdcd1^-/-^* P14 CD8^+^ T cells compared to WT (Fig. 7A). Assessing polyfunctionality, *Selplg^-/-^* P14 CD8^+^ T cells retained higher IFNγ^+^ TNFα^+^ frequencies while *Pdcd1^-/-^* and *Selplg^-/-^Pdcd1^-/-^* P14 CD8^+^ T cells had lower IFNγ^+^ TNFα^+^ frequencies relative to WT (Fig. 7B). When evaluating cytotoxic ability, we observed a small increase in the frequencies of IFNγ^+^CD107a^+^ *Selplg^-/-^* P14 CD8^+^ T cells compared to WT (Fig. 7C). By contrast, *Pdcd1^-/-^* and *Selplg^-/-^Pdcd1^-/-^* P14 CD8^+^T cells had significantly lower IFNγ^+^CD107a^+^ frequencies compared to WT (Fig. 7D). We observed no differences in granzyme B production in *Selplg^-/-^* or *Selplg^-/-^Pdcd1^-/-^* P14 CD8^+^ T cells but observed a small but significant increase in granzyme B production in *Pdcd1^-/-^* P14 CD8^+^ T cells, compared to their WT counterparts (Fig. 7D). Consistent with early timepoints, these findings suggested that while deleting PSGL-1 alone improves the maintenance of T-cell polyfunctionality, deleting PD-1 alone or in combination with PSGL-1 worsens T-cell function over the course of viral infection.

### The PD-1 pathway predominantly drives *Selplg^-/-^Pdcd1^-/-^* exhausted CD8^+^ T-cell accumulation

**Fig 8:**
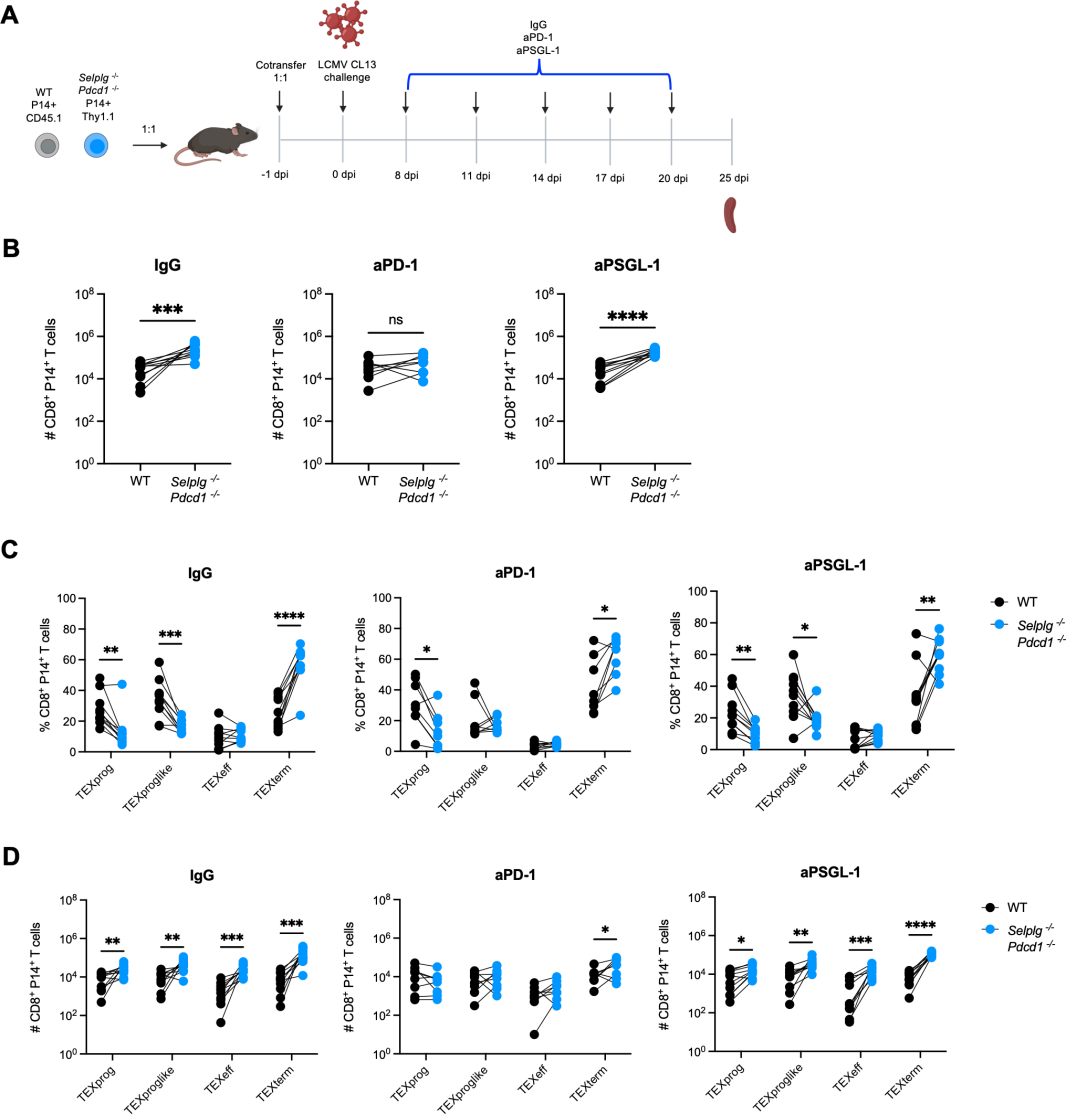
*Selplg^-/-^Pdcd1^-/-^* P14 CD8^+^ T cells accumulate due to PD-1 inhibition. (**A**) WT and *Selplg^-/-^Pdcd1^-/-^* P14 CD8^+^ T cells were adoptively co-transferred at a 1:1 ratio into WT recipients and infected with Cl13 one day after. Infected mice were subsequently treated with anti-IgG, anti-PD-1, or anti-PSGL-1 at 8, 11, 14, 17, and 20 dpi. Spleens were isolated at 25 dpi and WT and *Selplg^-/-^Pdcd1^-/-^* P14 CD8^+^ T cells were analyzed. (**B**) Numbers of WT and *Selplg^-/-^Pdcd1^-/-^* P14 CD8^+^ T cells after anti-IgG, anti-PD-1, or anti-PSGL-1 treatment. (**C**) Frequencies of SLAMF6^+^CX3CR1^−^ progenitor exhausted (TEXprog), CX3CR1^+^TIM3^−^ progenitor exhausted like (TEXproglike), CX3CR1^+^TIM3^+^ effector exhausted (TEXeff), and SLAMF6^−^CX3CR1^−^ terminal exhausted (TEXtem) between WT and *Selplg^-/-^Pdcd1^-/-^* P14 CD8^+^ T cells after anti-IgG, anti-PD1, or anti-PSGL-1 treatment. (**D**) Numbers of TEXprog, TEXproglike, TEXeff, and TEXtem between WT and *Selplg^-/-^Pdcd1^-/-^* P14 CD8^+^ T cells after anti-IgG, anti-PD1, or anti-PSGL-1 treatment. *P < 0.05, **P < 0.01, ***P < 0.001 (paired t-test). Data are representative of two combined independent experiments all with five or more mice per group (error bars, s.e.m.).

We next aimed to understand whether the accumulation of *Selplg^-/-^Pdcd1^-/-^* CD8^+^ T cells was driven by PD-1 or PSGL-1 inhibition. We adoptively co-transferred small numbers (1 × 10^3^) of WT P14 (CD45.1^+^) and *Selplg^-/-^Pdcd1^-/-^* (Thy1.1^+^) P14 CD8^+^ T cells into WT (CD45.2^+^Thy1.2^+^) hosts at a 1:1 ratio. One day later, mice were infected with Cl13. Mice were then treated with IgG, anti-PD-1, or anti-PSGL-1 at 8, 11, 14, 17, and 20 dpi. At 25 dpi, mice were sacrificed and co-transferred cells in the spleen were analyzed (Fig. 8A). As expected from our previous data, we observed increased numbers of *Selplg^-/-^Pdcd1^-/-^* P14 CD8^+^ T cells relative to WT cells in IgG-treated mice (Fig. 8B). Since *Selplg^-/-^Pdcd1^-/-^* P14 CD8^+^ T cells lack PSGL-1 and PD-1 expression, anti-PD-1 or anti-PSGL-1 did not change the numbers of *Selplg^-/-^Pdcd1^-/-^* P14 CD8^+^ T cells, in any of the treatment groups (Fig. 8B). However, in anti-PD-1-treated mice, WT P14 CD8^+^ T cells expanded to similar numbers as *Selplg^-/-^Pdcd1^-/-^* P14 CD8^+^ T cells compared to IgG-treated mice (Fig. 8B). We observed that WT P14 CD8^+^ T cells in anti-PSGL-1-treated mice did not increase and were similar as in IgG treated mice (Fig. 8B). We next evaluated the frequencies and numbers of exhausted T-cell subsets with the treatment groups. We observed that *Selplg^-/-^Pdcd1^-/-^* P14 CD8^+^ T cells were increased in frequencies and numbers for terminal exhausted subsets (Fig. 8C and D), and these subsets were increased in WT P14 CD8^+^ T cells after anti-PD-1 treatment but not anti-PSGL-1 treatment (Fig. 8C and D). Furthermore, anti-PD-1 treatment led to increased numbers of progenitor and terminal subsets in WT P14 CD8^+^ T cells, and these WT CD8^+^ T-cell subsets reached similar numbers as exhausted populations observed in the *Selplg^-/-^Pdcd1^-/-^* P14 CD8^+^ T cells (Fig. 8D). This antibody blockade data suggested that the PD-1 pathway was the major player in the increased and sustained maintenance of *Selplg^-/-^Pdcd1^-/-^* P14 CD8^+^ T cells and that PSGL-1 antibody blockade at late stages of infection has less impact on the numbers of virus-specific T cells than PSGL-1 genetic deletion.

## DISCUSSION

The expression of immune checkpoints is broadly associated with impairing T-cell function and the ability to successfully eliminate chronic antigens, such as chronic viral infections and cancers. Indeed, many studies have shown that blocking immune checkpoints using ICIs increased frequencies and improved the function of T cells, resulting in improved viral clearance and reduced tumor growth (13, 23, 24, 27, 28). Furthermore, simultaneous blockade of multiple immune checkpoints via combination ICI therapy resulted in additional improvement in T-cell function in murine models and extended survival of cancer patients (12, 13, 22, 23, 28). While current ICIs have demonstrated clinical success, many patients do not respond or develop resistance, requiring the identification of new immune checkpoint targets (16). PSGL-1 is a recently discovered immune checkpoint that negatively regulates the T-cell response (21, 22). Given that its deficiency improves T-cell function, PSGL-1 could be a promising new target. Previously, we found that the combination of PSGL-1 deletion with PD-L1 blockade at late stages of chronic viral infection resulted in increased anti-viral CD8^+^ and CD4^+^ T-cell frequencies with enhanced cytokine production (22). Consequently, we wanted to understand if this phenotype was T cell-intrinsic. In this work, we found that deletion of both PSGL-1 and PD-1 in anti-viral CD8^+^ T cells increased frequencies and improved the maintenance of these cells throughout the course of chronic infection. This suggests that relieving the “brakes” of these two immune checkpoints promotes the development of effector anti-viral T cells and that PD-1 and PSGL-1 combined act as negative regulators of the T-cell response. It is important to note that this sustained increase was also observed in CD8^+^ T cells deficient in PD-1 but not in CD8^+^ T cells deficient in PSGL-1, where CD8^+^ T-cell numbers and frequencies decreased. The increase in *Selplg^-/-^Pdcd1^-/-^* CD8^+^ T cells was primarily driven by PD-1 deficiency which increased their proliferation and survival. Cell-intrinsic PSGL-1 deficiency resulted in improved CD8^+^ T-cell function, as previously reported (5, 22). By relieving both PSGL-1 and PD-1 immune checkpoints, we also expected to see improved T-cell function. To our surprise, cell-intrinsic deficiency of PD-1 or both PD-1 and PSGL-1 resulted in drastically reduced CD8^+^ T-cell function in both the early and late stages of chronic viral infection. Like the patterns observed in changes in T-cell frequencies, this phenotype seemed to be primarily driven by PD-1 deficiency. The observed decrease in function of *Selplg^-/-^Pdcd1^-/-^* anti-viral CD8^+^ T cells was puzzling since PSGL-1 and PD-1 blocking antibodies either separate or combined have been shown to improve T-cell function (5, 22, 29). Consistent with our results, however, studies showed that *Pdcd1^-/-^* T cells had decreased cytokine production and cytotoxic potential in a chronic viral model (30). These differences between our results observed in a cell-intrinsic deletion model and those observed in blockade models are important for several reasons. First, this demonstrates that cell-intrinsic deletion of an immune checkpoint versus using monoclonal antibodies to block immune checkpoints can have very different impacts on T-cell development and response. Deleting a checkpoint may change T-cell differentiation during the response while blocking the checkpoint may just partially inhibit that response. Second, this highlights the utility of cell-intrinsic deletion models to understand the importance in the underlying biology that may only be partially observed in a blockade model. In our studies, while deletion of PD-1 was beneficial for increased numbers of anti-viral T cells, loss of PD-1 was detrimental to their function. Conversely, loss of PSGL-1 was detrimental to anti-viral T-cell persistence but was beneficial for their function. Based on this, both immune checkpoints play different T-cell intrinsic protective roles. While deletion of either immune checkpoint would not be beneficial, targeting these immune checkpoints using ICIs in a more temporary and dynamic way may be useful to optimize the anti-viral T-cell response.

Immune checkpoints have also been shown to regulate exhausted T-cell subset differentiation. As the exhaustion program proceeds, the increased expression of immune checkpoints marks the terminally exhausted fate. In our study, we found that deleting PSGL-1 maintained T cells in a more effector-like state but led to their overall decrease over time due to a terminal fate. By contrast, with PD-1 deletion, T cells proliferated and were maintained at higher levels than PSGL-1-deficient T cells, but these were more terminal and produced decreased cytokines on a per cell basis. The phenotypes observed in PD-1-deficient cells were replicated in PSGL-1/PD-1 dual deficient CD8^+^ T cells, demonstrating the dominance of the PD-1 pathway which limited proliferation and function in virus-specific T-cell exhaustion. While PSGL-1-deficient CD8^+^ T cell numbers were collectively fewer, PD-1 and PSGL-1/PD-1-deficient CD8^+^ T cells were increased, and these cells had a terminal rather than progenitor phenotype. In addition, while PD-1 and PSGL-1/PD-1-deficient CD8^+^ T phenotypically appeared more terminal, they had higher proliferation than WT or PSGL-1-deficient T cells, which sustained their numbers to late periods of chronic infection. Our findings combining PSGL-1/PD-1 deletion in T cells resulting in a terminal exhausted phenotype are consistent with studies showing that deleting the immune checkpoints CTLA-4, LAG-3, and PD-1 alone or in combination can alter phenotypes and functions of exhausted T cells (25, 31). These findings have important implications when thinking about types of therapies for patients with chronic diseases and persistent antigens. Understanding the mechanisms by which ICIs alter the balance of progenitor and terminally exhausted T cells, and the impact on T cell proliferation, survival, and function is critical to the development of more effective therapeutics.

Overall, our studies provide a better understanding of the roles that PD-1 and PSGL-1 immune checkpoints play on anti-viral CD8^+^ T cells during chronic viral infections. They reveal key opposing differences by which PSGL-1 and PD-1 pathways drive virus-specific T-cell exhaustion. The PD-1 pathway restricts the accumulation of exhausted virus-specific T cells but helps maintain their function, whereas PSGL-1 supports the maintenance of T cells but restricts their functional capacity. In addition, these studies provide novel insights into how immune checkpoints mediate the T-cell exhaustion program. Understanding the unique contributions of these immune checkpoints, and their potential blockade, provides critical insight into future treatment strategies. For example, ICIs are commonly mixed and co-delivered in combination treatment; however, this may not be the ideal strategy based on the observed T-cell response in some patients. Based on mechanisms elucidated herein, it could be of interest to test if primary treatment with anti-PD-1 or anti-PD-L1 could boost T-cell frequencies followed by secondary treatment with anti-PSGL-1 to boost T-cell function. Immune checkpoints play crucial roles in maintaining immune tolerance, preventing autoimmunity, and regulating immune responses against infections and cancer to prevent immunopathology (26, 32, 33). We observed yet another example of the importance of the protective roles of immune checkpoints on CD8^+^ T cells. There is a delicate balance between immune checkpoints being necessary but also detrimental to the T-cell response that must be considered. These findings enhance our understanding of inhibitory pathways in T cells, offering crucial insights that could lead to improved therapeutic strategies and immune checkpoint inhibitors for patients with chronic viral infections and cancer.

## MATERIALS AND METHODS

### Mice

C57BL/6J mice were purchased from Jackson Laboratory. P14 TCR transgenic mice were obtained from The Scripps Research Institute (originally from Dr. Charles D. Surh). These mice were bred to Ly5.1 (B6.SJL-Ptprc^a^ Pepc^b^/BoyJ) mice and Thy1.1 (B6.PL-Thy 1^a^/CyJ). *Selplg^-/-^* and *Pdcd1^-/-^* mice were purchased from Jackson Laboratory and bred with the P14 TCR transgenic mice in-house to generate the P14 *Selplg^-/-^* and P14 *Pdcd1^-/-^* mouse lines. P14 *Selplg^-/-^* and P14 *Pdcd1^-/-^* were bred to generate the P14 *Selplg^-/-^Pdcd1^-/-^* mouse line. All mice were bred in specific-pathogen-free (SPF) facilities and maintained in biosafety level 2 (BSL-2) facilities after infection in the vivarium at UC Irvine. Mice were genotyped by PCR prior to experiments to confirm genetic deletion. P14 TCR expression was confirmed by flow cytometry using antibodies against CD8, Vα2, and Vβ8. Male mice at 6–8 weeks of age were used. All experiments were approved by the animal care and use committees at UC Irvine (AUP-21–124).

### LCMV clone 13 (Cl13) virus production, tittering, and infections

All viral production, tittering, and dilution work were performed in BSL2-level biosafety cabinets.

BHK cells were plated in culture media (Dulbecco’s modified Eagle medium [DMEM] supplemented with 10% fetal bovine serum [FBS], 1% penicillin/streptomycin antibiotics, and 7% tryptose phosphate broth) at 5 × 10⁶ cells/20 mL per T175 flask for virus production. The next day when cells were ~60% confluent, cell media was aspirated and replaced with infection media (multiplicity of infection of 0.1, 10^6^ PFU/10^7^ cells). Flasks were incubated at 37°C for 1.5 hours with periodic shaking for even viral distribution. Infection media was then aspirated and replaced with 20 mL of fresh culture media, and cells were incubated for 48 hours. The supernatant was then collected and centrifuged at 2,200 rpm for 15 minutes at 4°C. Aliquots of the virus-containing supernatant were snap-frozen in dry ice and stored at −80°C. Additional 100 µL aliquots were prepared for tittering and quality control testing. BHK and Vero cells were generously provided by Dr. Elina Zuniga, UCSD.

Vero cells were plated at 0.25 × 10⁶ cells/mL in culture media (minimum essential medium [MEM] supplemented with 10% FBS, 1% penicillin/streptomycin antibiotics) in six-well plates and incubated for 24 hours until 90% confluent. Serial virus dilutions (10⁻²–10⁻⁷) were prepared in vero media and used to infect cells for 1 hour at 37°C as previously described. Infection media was then aspirated and replaced with an overlay of EMEM containing 0.5% agarose, 2% PSG, and 14% FBS. Plates were wrapped in tinfoil and incubated for 6 days at 37°C. Plaques were then fixed with 25% formaldehyde, stained with crystal violet, and counted. Viral titer was calculated as PFU/mL. The bulk viral stock was diluted to 1 × 10⁷ PFU/mL based on plaque assay results. Aliquots (1.1 mL) were snap-frozen and stored at −80°C. Before use in experiments, infection quality was confirmed in C57BL/6 mice. Mice were infected retro-orbitally (r.o.) with 2 × 10⁶ PFU of LCMV Cl13 in 200 µL. Immune responses were assessed at 8 and 30 dpi. This production and QC protocol ensures reproducibility and consistency in LCMV Cl13 production and subsequent applications.

For experiments, frozen viral stocks (1 × 10⁷ PFU/mL) were thawed and used to infect mice via r.o. injection with 200 µL stock solution containing 2 × 10^6^ plaque-forming units (PFUs) of LCMV Cl13. Once infected, mice were housed in BSL2-level facilities.

### P14 CD8^+^ T-cell enrichment and adoptive transfer

Spleens from naïve WT, *Selplg^-/-^*, *Pdcd1^-/-^*, and *Selplg^-/-^Pdcd1^-/-^* mice were isolated and processed according to the blood and spleen collection and processing protocol below. Cells were counted and resuspended at 100 × 10^6^ cells/mL in enrichment buffer (1X DPBS, 1% FBS) containing the following antibodies and dilutions (Biolegend: Fc block [1:30, clone 93], anti-CD4 [1:300, biotin, clone RM4-5], anti-CD19 [1:300, biotin, clone MB19-1], anti-CD11b [1:300, biotin, clone M1/70], anti-CD11c [1:300, biotin, clone N418], anti-CD24 [1:300, biotin, clone M1/69], anti-B220 [1:300, biotin, clone RA3-6B2]). Cells were incubated in this solution for 15 minutes at room temperature. 35 µL/mL of streptavidin-coated beads were added, suspensions were mixed, and incubated for an additional 5 minutes at room temperature. Cells were mixed and placed on a magnet (Stem Cell Technologies) for 5 minutes at room temperature. Supernatants containing purified CD8^+^ T cells were collected and subjected to an additional round of purification on the magnet. The final supernatant was collected and centrifuged at 300 × *g* for 5 minutes, and the pellet was resuspended in wash media (1× HBSS supplemented with 1% FBS, 2% penicillin/streptomycin antibiotics, 0.5% HEPES) for counting and QC check for purity. WT and either *Selplg^-/-^*, *Pdcd1^-/-^*, or *Selplg^-/-^Pdcd1^-/-^* P14 CD8^+^ T cells were transferred in equal numbers (1 × 10^3^) in 200 µL PBS into naïve C57BL/6 mice by r.o injection.

### Blood and spleen collection and processing

All blood and tissues were processed in BSL2-level biosafety cabinets. Blood was collected from mice at indicated timepoints. Briefly, mice were anesthetized and 100–200 µL of blood was collected retro-orbitally using heparin-coated capillaries (Fisher; 0266810) into 10 mL of RBC lysis buffer (pH 7.2 sterile solution composed of 450 mL 0.16M NH4Cl in water, pH 7.2 + 50 mL 0.17M TrisHCl, pH 8 in water). The blood was incubated for 45 minutes at room temperature and then centrifuged at 300 × *g* for 5 minutes. The supernatant was aspirated and the pellet was re-suspended in 10 mL wash media (1× HBSS supplemented with 1% FBS, 2% penicillin/streptomycin antibiotics, 0.5% HEPES). Cells were centrifuged at 300 × *g* for 5 minutes, the supernatant was aspirated, and the pellet was resuspended for analysis by flow cytometry.

Mice were euthanized to collect spleens at indicated timepoints. Briefly, spleens were macerated through a 70 µm strainer (Fisher; 22363548) using the rubber plunger of a sterile syringe. Strainers were washed with 10 mL wash media (1× HBSS supplemented with 1% FBS, 2% penicillin/streptomycin antibiotics, 0.5% HEPES). Cells were centrifuged at 300 × *g* for 5 minutes, the supernatant was decanted into bleach, and tubes were blotted. Cells were then resuspended in 1–2 mL of RBC lysis buffer (Sigma; R7757) and incubated for 1.5 minutes at room temperature. Lysis was quenched by adding 10 mL wash media (1× HBSS supplemented with 1% FBS, 2% penicillin/streptomycin antibiotics, 0.5% HEPES). Cells were centrifuged at 300 × *g* for 5 minutes, the supernatant was decanted into bleach, and tubes were blotted. Cells were then resuspended and counted.

### Flow cytometry

For cell surface staining, 2 × 10^6^ cells were incubated in a staining buffer (1× DPBS supplemented with 0.5% FBS and 0.05% 10% NaN_3_ in 1× DPBS) for 20 minutes at 4°C containing antibodies for expression of surface proteins. For intranuclear transcription factor staining, cells were fixed and permeabilized using the Invitrogen Foxp3/transcription factor fixation/permeabilization kit (Fisher; 50–112-9060) and stained in permeabilization buffer according to the manufacturer’s protocol. For functional assays, 2 × 10^6^ cells from infected mice were cultured for 4 hours at 37°C with 2 μg/mL of GP_33-41_ peptides (AnaSpec; AS-61669in T cell media (RPMI-1640 containing 10 mM HEPES, 1% non-essential amino acids and L-glutamine, 1 mM sodium pyruvate, 10% heat-inactivated FBS, and penicillin/streptomycin antibiotics) containing brefeldin A (1 µg/mL; Sigma-Aldrich), and IL2 (50 u/mL). The cells were then stained with antibodies for expression of surface proteins, fixed and permeabilized using the BD Cytofix/Cytoperm kit (Fisher; BDB554714), and stained with antibodies for intracellular cytokine detection. To evaluate cell degranulation, 2 × 10^6^ cells from infected mice were cultured for 4 hours at 37°C in T-cell media (RPMI-1640 containing 10 mM HEPES, 1% non-essential amino acids and L-glutamine, 1 mM sodium pyruvate, 10% heat-inactivated FBS, and penicillin/streptomycin antibiotics) with anti-CD107α. Cells were always stained in 50 µL volumes. The following antibodies and dilutions were used in this study: Biolegend CD8 (1:200, BV785, clone 53–6.7), Vα2 (1:200, PE, clone B20.1), Thy 1.1 (1:200, APC, clone OX-7), CD45.1 (1:200, PE-Cy7, clone A20), CD107α (1:200, FITC, clone 1D4B), IFNγ (1:100, APC, clone XMG1.2), TNFα (1:100, PE-Cy7, clone MP6-XT22), TIM-3 (1:200, BV605, clone RMT3-23), KLRG-1 (1:200, FITC, clone 2F1), CX3CR1 (1:200, BV510, clone SA011F11), CD95/FAS (1:200, PE, clone SA367H8), TIGIT (1:200, BV421, clone 1G9), LAG-3 (1:200, BV605, clone C9B7W), Cell Signaling Technology TCF-1 (1:100, Alexafluor488, C63D9), Miltenyi Biotec TOX (1:100, PE, clone REA473), BD Biosciences Vb8.1.2 (1:200, FITC, clone MR5-2), SLAMF6 (1:200, BV421, clone 13G3), Invitrogen Granzyme B (1:50, PE, clone GB12), and CD101 (1:200, PE, clone Moushi101).

### Gating strategy and calculations

For all flow cytometry panels, the following gating strategy was applied: lymphocytes, single cells (by forward scatter), single cells (by side scatter), total CD8^+^ cells, and then CD45.1^+^ for WT P14 or Thy1.1^+^ for *Selplg^-/-^*, *Pdcd1^-/-^*, or *Selplg^-/-^Pdcd1^-/-^* CD8^+^ P14 T cells. Within co-transferred populations additional markers were analyzed and FMOs were used for gate placement.

For CD8^+^ T-cell exhaustion populations, co-transferred cells were further visualized with SLAMF6 by CX3CR1. Progenitor T cells (TEXprog) were SLAMF6^+^CX3CR1^-^ and terminal T cells (TEXterm) were SLAMF6^−^CX3CR1^−^. The CX3CR1^+^ population was further analyzed for TIM-3 expression, where progenitor-like cells (TEXproglike) were CX3CR1^+^TIM-3^−^ and effector cells (TEXeff) were CX3CR1^+^TIM-3^+^.

Percent ratios of CD8^+^ P14 T cells were calculated by taking the frequency of either individual transferred population within the CD8^+^ gate and dividing by the added frequencies of total co-transferred populations within CD8^+^ T cells, then multiplying by 100.

Absolute numbers of P14 CD8^+^ T cells in the spleen were calculated by taking the frequency of the desired population within the total single-cell gate, dividing by 100, and multiplying by the total live cell spleen count.

### *In Vivo* antibody treatments

Naïve WT and *Selplg^-/-^Pdcd1^-/-^* P14 CD8^+^ T cells were isolated from mouse spleens by magnetic sorting (Stem Cell Technologies, using negative selection) in accordance with the manufacturer’s protocol. WT and *Selplg^-/-^Pdcd1^-/-^* P14 T cells were transferred in equal numbers (1 × 10^3^ P14) in 200 µL PBS into naïve C57BL/6 mice by retroorbital (r.o) injection. One day later, WT mice were infected with 2 × 10^6^ PFU LCMV Cl13 by r.o. injection. Subsequently, 200 µg (200 µL PBS) of anti-PD-1 (clone RMP1–14), anti-PSGL-1 (clone 4RA10), or rat IgG (Sigma; I4131) isotype control was administered by intraperitoneal (i.p) injection five separate times in WT mice. Antibody injections started at 8 days post-Cl13-infection and continued every 3 days until 20 days post-infection (8, 11, 14, 17, and 20 dpi). *In vivo* mAbs were purchased from BioXcell (New Hampshire, USA). Mice were euthanized for spleen collection at 25 days post-infection.

### Data analysis

Flow cytometry data were analyzed using FlowJo software. Graphs were made using GraphPad Prism software. GraphPad Prism was used for statistical analysis to compare outcomes using a two-tailed paired t-test. Significance was set to *P* < 0.05 and represented as *<0.05, **<0.001, ***<0.005, and ****<0.0001. Error bars show the standard error of the mean (s.e.m.).

## Data Availability

The original contributions presented in the study are included in the article and its supplemental material. Further inquiries can be directed to the corresponding author.

## SUPPLEMENTAL MATERIAL

Supplemental figures jvi.02242-24-s0001.pdf Figures S1 to S3.

ASM does not own the copyrights to Supplemental Material that may be linked to, or accessed through, an article. The authors have granted ASM a non-exclusive, world-wide license to publish the Supplemental Material files. Please contact the corresponding author directly for reuse.
