# Supplementary material for: Contrasting roles of PSGL-1 and PD-1 in regulating T-cell exhaustion and function during chronic viral infection
Source: J Virol. 2025 Feb 6;99(3):e02242-24. doi: 10.1128/jvi.02242-24 (PMC11915808; doi:10.1128/jvi.02242-24)
Supplement: Supplemental figures — Figures S1 to S3. [file jvi.02242-24-s0001.pdf]

Supplementary Figure 1

A

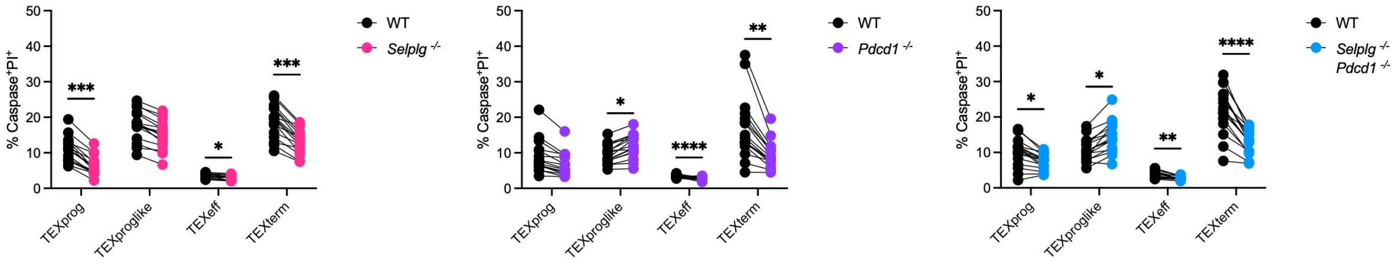

B

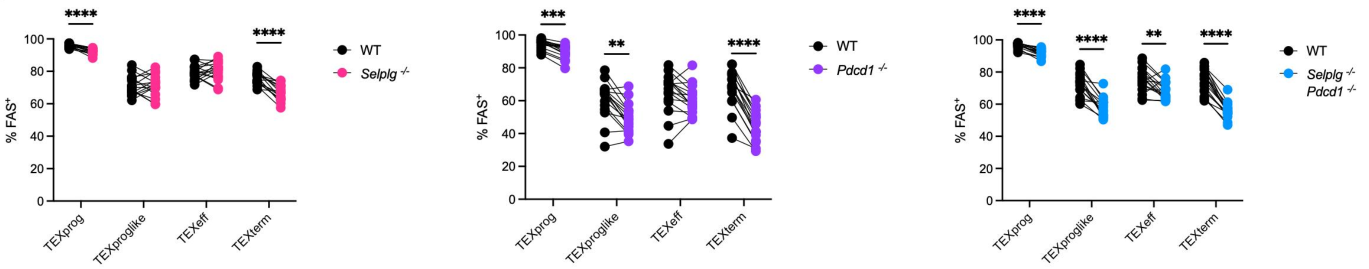

Supplementary Figure 2

A

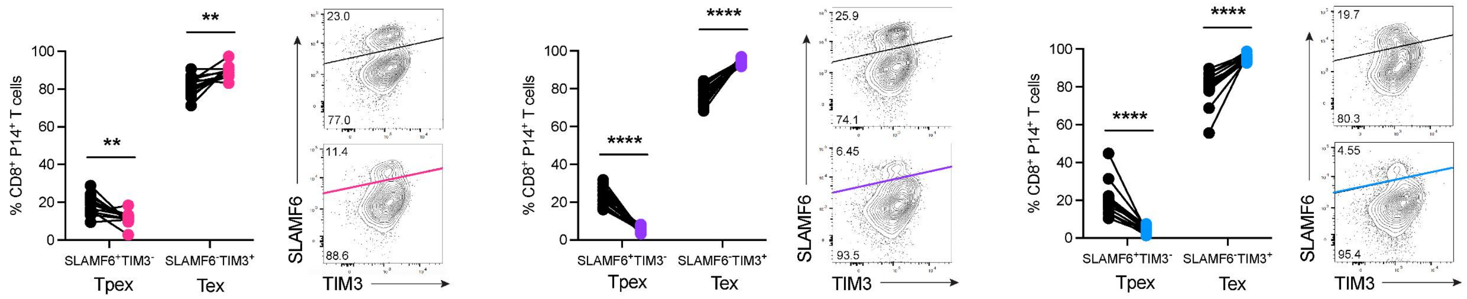

B

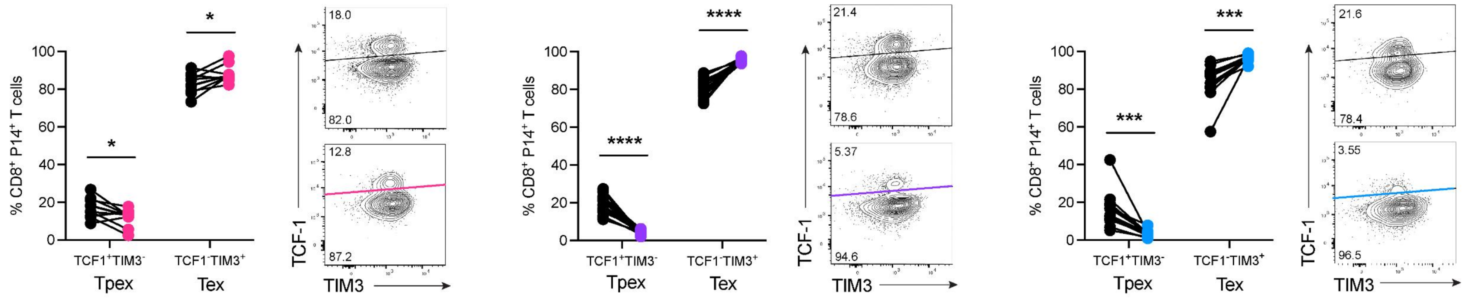

Supplementary Figure 3

A

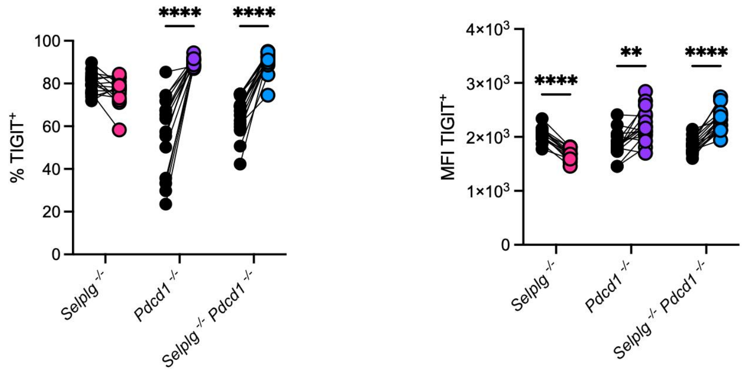

B

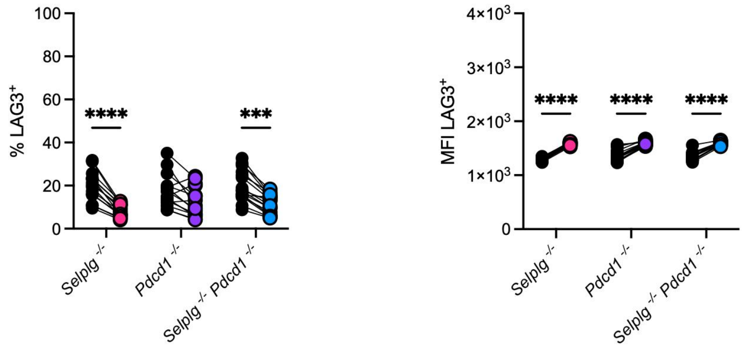

C

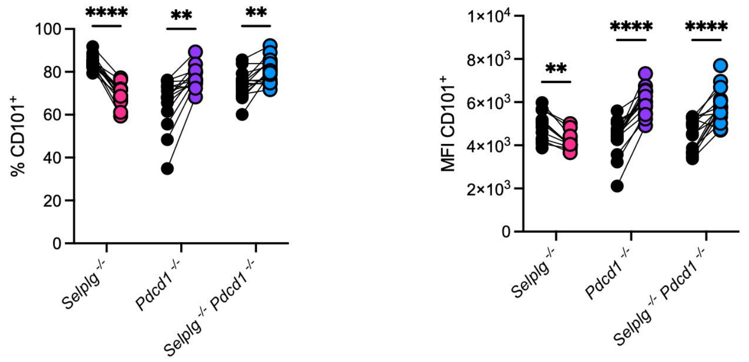

Supplementary Figure 1. Expression of survival markers in exhausted subsets of co-transferred *Selplg$^{-/-}$Pdcd1$^{-/-}$* P14 CD8^{+} T cells. Spleens were isolated from Cl13 infected mice at 12dpi. **A)** Frequencies of Caspase^{+}PI^{+} cells within SLAMF6^{+}CX3CR1^{-} progenitor exhausted (TEXprog), CX3CR1^{+}TIM3^{-} progenitor exhausted like (TEXproglike), CX3CR1^{+}TIM3^{+} effector exhausted (TEXeff), and SLAMF6^{-}CX3CR1^{-} terminal exhausted (TEXtem) between WT and either *Selplg$^{-/-}$*, *Pdcd1$^{-/-}$*, or *Selplg$^{-/-}$Pdcd1$^{-/-}$* P14 CD8^{+} T cells. **B)** Frequencies of FAS^{+} cells within TEXprog, TEXproglike, TEXeff, and TEXtem between WT and either *Selplg$^{-/-}$*, *Pdcd1$^{-/-}$*, or *Selplg$^{-/-}$Pdcd1$^{-/-}$* P14 CD8^{+} T cells. *p < 0.05, **p < 0.01, ***p < 0.001, (paired t-test). Data are representative of two combined independent experiments all with five or more mice per group (error bars, s.e.m.).

Supplementary Figure 2. *Selplg$^{-/-}$Pdcd1$^{-/-}$* P14 CD8^{+} T cells have decreased Tpex cells during chronic viral infection. Spleens were isolated from Cl13 infected mice at 30dpi. Frequencies and representative flow cytometry plots of Tpex and Tex cells characterized by (**A**) SLAMF6 and TIM3 or (**B**) TCF1 and TIM3 between WT and either *Selplg$^{-/-}$*, *Pdcd1$^{-/-}$*, or *Selplg$^{-/-}$Pdcd1$^{-/-}$* P14 CD8^{+} T cells. *p < 0.05, **p < 0.01, ***p < 0.001, (paired t-test). Data are representative of five independent experiments all with five or more mice per group (error bars, s.e.m.).

Supplementary Figure 3. Expression of terminal exhaustion markers within co-transferred P14 CD8^{+} T cells during chronic viral infection. A) Spleens were isolated from Cl13 infected mice at 28dpi. Frequencies (left) and mean fluorescence Intensity (right) of immune checkpoint markers **(A)** TIGIT **(B)** LAG-3 and **C)** CD101 between WT and either *Selplg$^{-/-}$*, *Pdcd1$^{-/-}$*, or *Selplg$^{-/-}$Pdcd1$^{-/-}$* P14 CD8^{+} T cells. *p < 0.05, **p < 0.01, ***p < 0.001, (paired t-test). Data are

representative of two combined independent experiments all with five or more mice per group (error bars, s.e.m.).
